# The Medical Care of Children in Elementary Schools

**Published:** 1903-12-26

**Authors:** 


### The Medical Care of Children in Elementary Schools.

To those interested in social and educational questions, and indeed to every lover of his country, the facts contained in a report on the physical condition of children attending the Board Schools of Edinburgh and Aberdeen which was issued at the end of last year must have carried a painful shock. It will be remembered that Dr. Mackenzie, the author of the report in question, regarded the conditions in Aberdeen as generally satisfactory. But in Edinburgh, on the other hand, there existed widespread evidences of physical deterioration, and among the children drawn from the poorest quarters of the city the state of matters in relation to food supplies, clothing, personal cleanliness, and health was simply appalling. More recently the Army authorities have drawn official attention to the unsatisfactory condition of the physique and development of men offering themselves as recruits, and as a result of this the Government has appointed a committee to consider whether the whole question of the physical condition of the people should not be referred to a Royal Commission. From a recent article by Sir John Gorst it may be concluded that there are those who are prepared to advocate a more expeditious procedure than that offered by a Royal Commission, and, whether one plan or the other is adopted, it may be taken as certain that the question of the physical condition of the children in attendance at elementary schools will be shortly pushed to the front. Even the current fiscal controversy is contributing something to this end by emphasising the tendency of a free-trade policy to crowd the towns at the expense of the country districts. For members of the medical profession the question here referred to has a threefold interest. In the first place it concerns them as citizens who, equally with their fellows, desire the well-being and efficiency of the rising generation and the ability of the country to maintain its position in the keen struggle of modern international life. Secondly, the medical practitioner by his education and training is specially qualified to appreciate both the importance of vigorous physical health and the influence of the conditions necessary to promote this. Hence it may be fairly said that there lies upon him a special responsibility in excess of that which rests on the ordinary citizen. His position gives him the opportunity to exercise an educative influence on the general community and this may reasonably be included in the claims of good citizenship. The third relationship of the medical profession to the physical conditions of children attending the elementary schools arises from an important if somewhat less elevated consideration. It may be taken as certain that in proportion as the inspection of the children becomes thorough the need for medical assistance will increase. This will not only apply to the methods and tests adopted for purposes of examination. It will also extend to questions of treatment. An illustration of what may be expected has been afforded during the last year or two by the practice of the School Board of London in regard to the sight testing of the school children. After a preliminary rough test enormous numbers of these children have been condemned as the subjects of defective sight and their parents have been en-encouraged to make themselves recipients of charity by attendance on public hospitals. In this way the burden of time and labour placed upon the profession without recognition or reward has been very great. It cannot be doubted that a similar experience is probable in many other departments of medical practice. All this it seems to us is most unfair. If it is right and proper that school children should be medically examined and treated without expense to their parents this should be undertaken at the charge of the whole community and not of a limited class. What is necessary or wise as a matter of public policy ought to be a charge on public funds, and the medical practitioner, equally with other citizens, must contribute his share. Similarly the claims of benevolence have for him the same force as they have for others. But neither public charges nor private charity ought to be urged in unfair proportion on any section of the community, and whilst the medical profession is not deaf to either of these claims it should take care to protect itself against any further attempt to increase unfairly its share of public burdens.

